# Laboratory Radiometric Calibration Technique of an Imaging System with Pixel-Level Adaptive Gain

**DOI:** 10.3390/s23042083

**Published:** 2023-02-13

**Authors:** Ze Li, Jun Wei, Xiaoxian Huang, Feifei Xu

**Affiliations:** 1 Shanghai Institute of Technical Physics, Chinese Academy of Sciences, Shanghai 200083, China; 2 School of Electronic, Electrical and Communication Engineering, University of Chinese Academy of Sciences, Beijing 100049, China; 3 Key Laboratory of Infrared System Detection and Imaging, Chinese Academy of Sciences, Shanghai 200083, China

**Keywords:** pixel-level adaptive gain, high dynamic range, high sensitivity, laboratory radiometric calibration

## Abstract

In a routine optical remote sensor, there is a contradiction between the two requirements of high radiation sensitivity and high dynamic range. Such a problem can be solved by adopting pixel-level adaptive-gain technology, which is carried out by integrating multilevel integrating capacitors into photodetector pixels and multiple nondestructive read-outs of the target charge with a single exposure. There are four gains for any one pixel: high gain (HG), medium gain (MG), low gain (LG), and ultralow gain (ULG). This study analyzes the requirements for laboratory radiometric calibration, and we designed a laboratory calibration scheme for the distinctive imaging method of pixel-level adaptive gain. We obtained calibration coefficients for general application using one gain output, and the switching points of dynamic range and the proportional conversion relationship between adjacent gains as the adaptive-gain output. With these results, on-orbit quantification applications of spectrometers adopting pixel-level automatic gain adaptation technology are guaranteed.

## 1. Introduction

### 1.1. The Necessity to Expand Dynamic Range

Since the late 20th century, research on spaceborne imaging spectrometers has been globally carried out. Representative imaging spectrometers are the moderate-resolution imaging spectrometer (MODIS) and subsequent visible infrared imaging radiometer (VIIRS) developed by NASA, and the medium-resolution imaging spectrometer (MERIS) and subsequent ocean and land color instrument (OLCI) developed by ESA. All of them provide a large amount of remote-sensing monitoring data with high sensitivity and stable performance for water and land observation. However, the limit of the core device manufacturing process may lead to a limited dynamic range of the spectrometer, which impacts environmental monitoring. For example, U.S.-developed MODIS is easily susceptible to data saturation in near-shore coastal Case 2 waters with high reflectivity when there are water blooms on the ocean surface [1].

With the improvement of user application requirements, the design of imaging spectrometers is more inclined towards the diversification of monitoring scenes and the generalization of data. The Pre-Aerosol Clouds and Ocean Ecosystem (PACE), a preresearch aerosol, cloud, and ocean ecosystem monitoring tool currently used in the United States, aims to explore the relationship between global climate change and the carbon cycle through multiscene observations [2,3,4]. However, the difference among the entrance pupil radiance of terrestrial targets can reach 120 dB; for example, the reflectance of weak signals such as a class of water bodies in the ocean does not exceed 5%, and the irradiance of thick clouds and sea ice is close to one solar constant [5,6]. Under the requirement of comprehensive remote sensing monitoring, how to extend the dynamic range of an observation system while maintaining high signal sensitivity is a research challenge.

### 1.2. Limitations on Extending Dynamic Range

The current methods for dynamic range extension include the following: applying digital micromirror devices (DMD) or optical modulation devices such as reflective liquid crystal light valves to change the optical system’s light intensity [7] with the disadvantage that multiple imaging adjustments are required, but not suitable for imaging spectrometers [8]; using single-detector multiple-exposure or multidetector single-exposure methods to fuse low-dynamic-range images of different exposure levels into high-dynamic-range images, although single-detector multiple-exposure methods are difficult to apply to motion scenes, while multidetector single-exposure methods have a complex and large structure at a very high cost [9]; the expansion of the dynamic range of push broom imaging spectrometers to improve photodetection and subsequent electronic links by using a logarithmic-response pixel circuit, horizontal gate overflow technology, and frame gain switching technology.

For a single-gain imaging mode, the dynamic range (DR) can be expressed as in Formula (1):

(1) $$DR=\frac{DN_{\max}}{DN_{noise}},$$

where DR is the dynamic range, $DN_{\max}$ is the maximal signal digital number (DN), and $DN_{noise}$ is the DN of noise.

Due to the fabrication process, the upper limit of the CMOS detector voltage value is limited by the supply voltage. In addition, the quantization elements of ADC cannot be improved due to the limitation of channel capacity. The only way to improve the dynamic range of a detector is to improve the full-well capacity of the detector. The relationship between the full-well capacity of the system and the quantization value after analog-to-digital DN conversion is shown in Formula (2); the relationship between the signal charge and the signal DN is shown in Formula (3):

(2) $$DN_{\mathrm{sat}\;}=G\cdot Q_{FW},$$

(3) $$DN_{\mathrm{signal}\;}=G\cdot Q_{\mathrm{signal}\;}$$

where $DN_{\mathrm{sat}\;}$ is the saturated DN, $Q_{FW}$ is the full-well capacity, $DN_{\mathrm{signal}\;}$ is the signal DN, $Q_{\mathrm{signal}\;}$ is the signal charge, and *G* is the gain of the imaging system.

Since the number of ADC quantization bits is determined, quantized saturation value $DN_{sat}$ is also fixed. Gain *G* must be reduced in order to expand the full-well capacity $Q_{FW}$. According to Formula (3), reducing gain *G* causes signal electron number $DN_{signal}$ to decrease simultaneously, but output noise $DN_{noise}$ is determined in the steady state of the detector, and the signal-to-noise ratio (SNR) is greatly reduced under a low-radiance input condition. The SNR equation is shown in Formula (4):

(4) $$SNR=\frac{N_{\min}}{\sqrt{N_{\min}+N_{\mathrm{noise}\;}^{2}}}$$

where $N_{\min}$ is the minimal signal charge that could be detected by the system, and $N_{noise}$ is the noise charge. Usually, image quantization details are insufficient when the SNR is less than 100. Therefore, it is difficult to achieve a high dynamic range and high sensitivity using CMOS detectors in single-gain mode.

### 1.3. The Advantage of Pixel-Level Adaptive Gain and Difficulties Encountered in Radiometric Calibration

Generally, multiple gains are set to ensure a high SNR and expand the dynamic range. For example, the SeaWiFS developed by the U.S. uses multiple detectors with different gains to image under the same spectral channel [10]; the VIIR has a day–night band (DNB) that uses three different gains to observe targets under different lighting conditions during morning, evening, and night [11,12,13]. On-orbit payloads with multiple gains generally use whole-row or full-frame gain to switch, which still produces signal saturation locally and impacts the monitoring of some regions with large variations.

The pixel-level adaptive-gain imaging system integrates four gains into one pixel in order to expand the dynamic range. The gains are high gain (HG), medium gain (MG), low gain (LG), and ultralow gain (ULG). A multistage integrating capacitor is integrated into a single pixel of the CMOS detector, and a single exposure is performed during push-broom imaging with high-speed movement relative to the ground. Since the quantum efficiency of the detector is a fixed value, the total number of photocharges after exposure remains unchanged. The relationship among capacitance values, photocharges, and output voltage values is shown below:

(5) $$C=\frac{Q}{U}$$

where *C* is the capacitance value, *Q* is the photocharges, and *U* is the output voltage value.

Formula (5) shows that, when photocharge *Q* is constant, the capacitance value *C* of the integrating capacitor is larger, the output voltage value is lower, and the ADC quantization bit number is constant; therefore, the output detector DN is less likely to be saturated. The photocharge after exposure is read out with capacitors of different capacitance values.

The laboratory calibration of satellite instruments usually includes spectral calibration, relative radiometric calibration, and absolute radiometric calibration. In satellite remote sensing, the obvious fringe caused by an inconsistent response of a push-type sensor detector reduces the image quality. The purpose of relative radiation calibration is to calibrate the inconsistency of detector responses, so as to eliminate the horizontal fringe of the detector [14]. Absolute radiometric calibration is the process of correlating instrument-measured values (DN) with absolute physical quantities such as spectral radiance. Accurate radiometric calibration is the basis for all quantitative applications of imaging systems [15]. The relationship between the radiance of a ground object and the measured value of an instrument is usually designed to be linear, and the parameters in the linear equation need to be solved through laboratory radiometric calibration.The quantitative relationship between radiance and photodetector DN in absolute radiometric calibration in the laboratory is expressed with Formula (6):

(6) L(λ)=Gain∗DN+Bias,

where L(λ) is the radiance at the entrance pupil in units of mW · cm${}^{-2}\;\cdot \;$sr${}^{-1}\;\cdot \;\mu$m${}^{-1}$, DN is the observation DN of the photodetector, Gain is the absolute radiometric calibration coefficient, Bias is the absolute radiometric calibration intercept, and the units of Gain and Bias are the as same as L(λ).

Absolute radiometric calibration systems usually include the following: standard irradiance lamp-integrating sphere radiometric calibration, standard detector-integrating sphere radiometric calibration, spectral radiometer-monochromatic parallel light radiometric calibration, and standard detector-diffuser radiometric calibration [16]. Standard detector-integrating sphere radiometric calibration greatly improves the accuracy of radiation measurements and greatly reduces uncertainty in comparison to other methods [16], so we adopted this method and used the feature spectrometer (ASD) as the standard detector.

The characteristics of a pixel-level adaptive-gain imaging system, such as high sensitivity and high dynamic range, mean that it is considerably different from other instruments in laboratory radiometric calibration and related parameter determination. Generally, push-scan imagers have only one or two gains, and only two sets of linear equation coefficients are measured. Since there are four gains, the corresponding coefficients of four groups of linear equations should be determined. In the process of laboratory radiometric calibration, we need to find the linear dynamic range of four gains. It is also a new challenge to find the switching point and establish the relationship among the four gains during gain switching. We designed a laboratory calibration system, build on the principle of pixel-level adaptive-gain imaging mode, and analyze laboratory radiometric calibration requirements and designs. Lastly, the corresponding experimental conclusions are provided.

## 2. Pixel-Level Adaptive-Gain Imaging System and Its Laboratory Radiometric Calibration System

### 2.1. Introduction of Pixel-Level Adaptive-Gain Imaging System

The pixel-level adaptive-gain imaging system studied in this paper was mounted onto a medium-resolution imaging spectrometer with a field angle of over 64${}^{\circ }$. It is mainly used for detecting the ocean color of offshore coastal zones and inland lakes with high sensitivity and high precision. It combines land, atmosphere, and cirrus cloud detection. It uses a customized back-illuminated thinned CMOS detector. The detector pixel size is 2176×320, where 2176 is the number of pixels in the spatial dimension, and 320 is the number of pixels in the spectral dimension. The spectral range of visible light is 375–950 nm. The spectral sampling interval of a single row of pixels is 2.16 nm, and the half-power bandwidth is about 2.5 nm.

The push-broom imaging system with pixel-level adaptive gain can provide multiple nondestructive read-outs for the target charge with single exposure by setting four gains in a single pixel. After the pixel selects the appropriate gain, the target information is transmitted, thereby meeting the comprehensive requirements of a high SNR without saturation.

**Figure 1:**
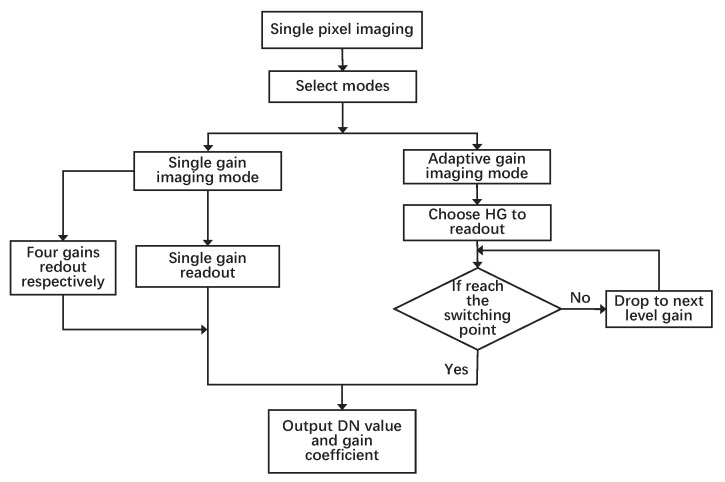
Flowchart of single-pixel imaging mode.

There are two intrapixel imaging modes, namely, single- and adaptive-gain imaging modes. The imaging process in a single pixel is shown in Figure 1. Single-gain imaging is used for imaging in a specific scene with similar reflectivity or for scaling the coefficient calibration of two adjacent gains. In this mode, signals can be nondestructively read out multiple times with only an initial reset because of the detector’s nondestructive read-out (NDRO) technology. All the pixels on the detector are read out by the same capacitor of four multistage integrating capacitors or all pixels can be read out by four capacitors to obtain all the data of the four gains for the same target.

In adaptive-gain imaging mode, data are first read from the highest-gain HG. If the output DN reaches the HG saturation value at this time, the lower-gain MG is selected for data reading and so on until the selected gain can read out the unsaturated signal. In this mode, only the linear region of each gain is used. When the current gain output DN exceeds the maximal linear dynamic range, it enters the linear region of the next gain to read the data.

Theoretically, signal saturation does not occur in pixel-level adaptive-gain imaging mode. The principle is to retain a high-gain image for the details of the dark part, but the details of the bright part and the high-gain signal are all saturated (lost); therefore, the low-gain data of the corresponding pixel are used to restore the image. After obtaining the whole image, the adaptive-gain image needs to be fused. The high dynamic image relationship after four gain fusions is shown in Equation (7):

(7) $$Y_{HDR}=\{\begin{array}{c} YH\left(YH\leq SW_{HG}\right)\\ K_{HM}YM-B_{HM}\left(YH>SW_{HG},YM\leq SW_{MG}\right)\\ K_{HL}YL-B_{HL}\left(YM>SW_{MG},YL\leq SW_{LG}\right)\\ K_{HU}YU-B_{HU}\left(YU\leq SW_{ULG}\right) \end{array}$$

where YH, YM, YL, and YU are the output DNs of the four gains; $K_{HM}$, $K_{HL}$ and $K_{HU}$ are the proportional conversion coefficients between HG and MG, HG and LG, HG and ULG, respectively; $B_{HM}$, $B_{HL}$, and $B_{HU}$ are the corresponding conversion offsets. $SW_{HG}$ is the DN of the switching point of the HG. Similarly, $SW_{MG}$, $SW_{LG}$, and $SW_{ULG}$ are the DNs of the switching points corresponding to the three remaining gains.

**Figure 2:**
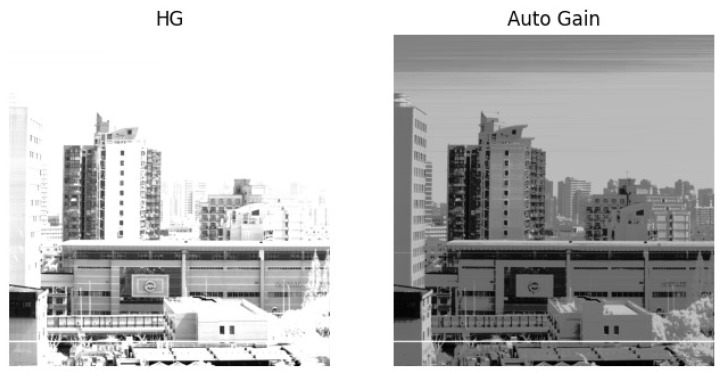
Comparison of HG imaging and adaptive-gain imaging under the same conditions.

Figure 2 shows that HG imaging under clear weather produces a large amount of data saturation, while an adaptive-gain image under the same imaging conditions retains complete image information.

### 2.2. Analysis of Laboratory Radiation Calibration Requirements

From the previous section, we understand that a pixel-level adaptive-gain imaging system is an optical payload that takes into account both high SNR and high dynamic range. To realize its high-precision quantitative application, it is necessary to fully determine the calibration parameters before launch. Usually, the laboratory calibration of conventional remote-sensing instruments includes dark-current measurement, an absolute radiation calibration coefficient, nonlinearity, SNR, and dynamic-range determination.

**Figure 3:**
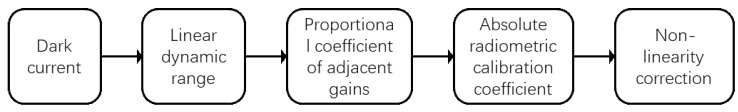
Flowchart of the laboratory calibration of an adaptive-gain imaging system.

However, a pixel-level adaptive-gain imaging system integrates four gains into one pixel and switches adaptively. It is necessary to separately measure the linear dynamic range of each gain to determine the switching-point DN of adjacent gain. After determining the linear range of each gain, it is necessary to determine the proportional coefficient between adjacent gains in order to unify the data of all pixels after adaptive-gain imaging. In addition, the output DNs at different energy levels of the integrating sphere should be used to determine the absolute radiation calibration coefficients of the four gains to establish the relationship between the detector response and the energy of the entrance pupil. Because the single-gain imaging mode has application requirements in specific scenarios, it is necessary to correct the region with severe nonlinearity of single-gain imaging. The required laboratory calibration process is shown in Figure 3.

### 2.3. Design of Laboratory Radiation Calibration System

**Figure 4:**
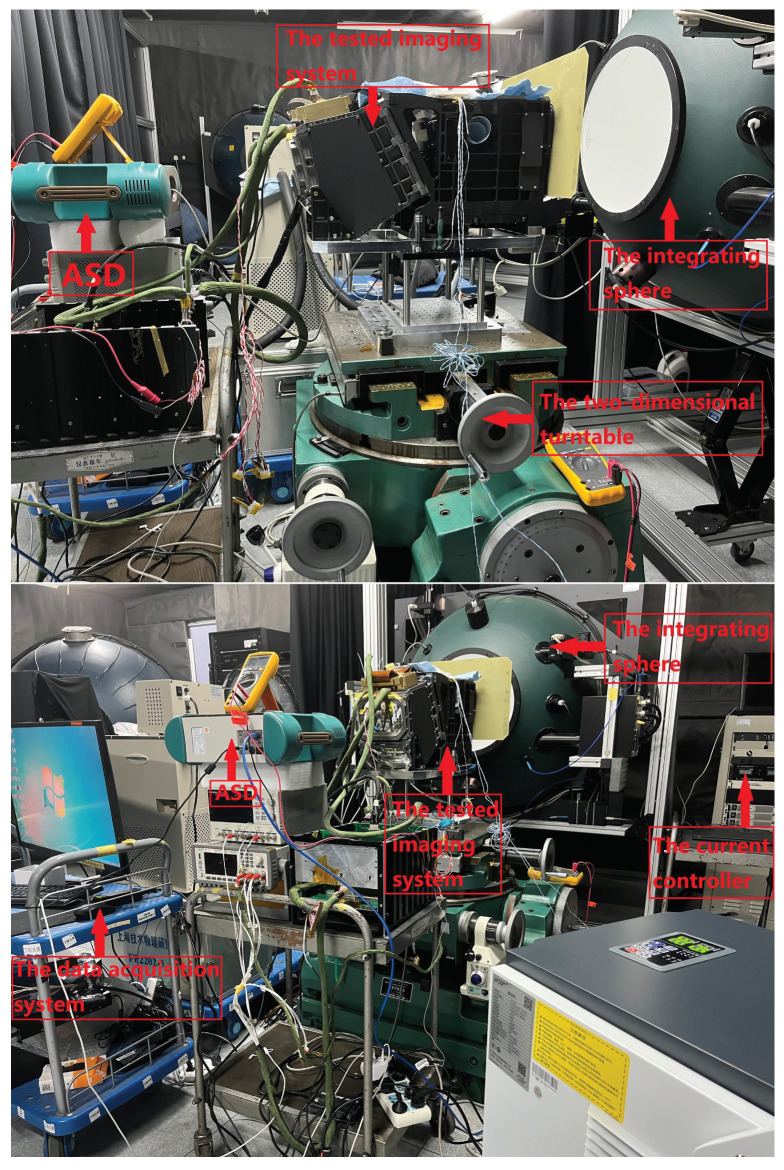
Laboratory radiation calibration system.

Generally, a spectrometer, whether it is dispersion- or interference-type, uses the integrating sphere close-range observation method for laboratory radiometric calibration. The test equipment includes an integrating sphere that outputs stable and uniform light, a current controller that adjusts the output light intensity of the integrating sphere, an ASD as a reference detector, a two-dimensional turntable that adjusts the pitch angle and azimuth angle between the detector surface and the integrating sphere, and a data acquisition system. During the test, the output energy of the integrating sphere is regulated by the current controller, and the data acquisition equipment is used to monitor and obtain the response data of the photodetector at different gains under different energies. The laboratory calibration system is shown in Figure 4.

**Figure 5:**
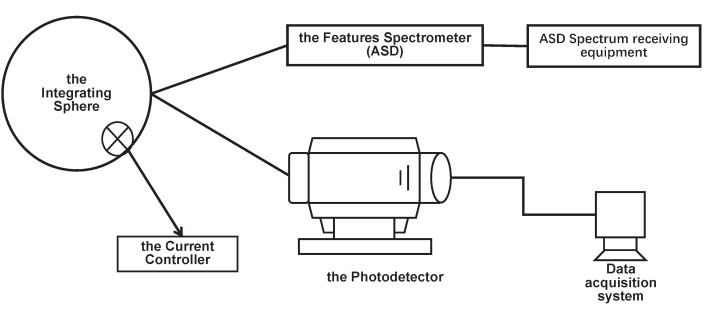
Experimental design diagram for the determination of the absolute radiometric calibration coefficient.

The radiometric calibration system of a pixel-level adaptive-gain imaging system obtains uniform incident light by approaching the integrating sphere at a close distance and synchronously detects the output energy of the integrating sphere with ASD. The optical fiber probe of an ASD almost coincides with the optical inlet of the imaging system being measured, so the radiance difference caused by distance is not considered. The design diagram of the experimental absolute radiometric calibration coefficient system is shown in Figure 5.

## 3. Laboratory Radiation Calibration Process Details and Results

This section describes the process and details of the laboratory radiometric calibration of a pixel-level adaptive-gain imaging system, and gives the experimental results and calibration coefficients.

### 3.1. Dark-Current Determination

Dark current is the output value of the photodetector when the rated voltage is applied without a light incident. The dark current limits the minimal luminous flux that the photodetector can measure, and brings false signals and noise [17]. The dark-current test is used to determine the minimal luminous flux of the photodetector, which is a necessary item for laboratory calibration testing.

**Figure 6:**
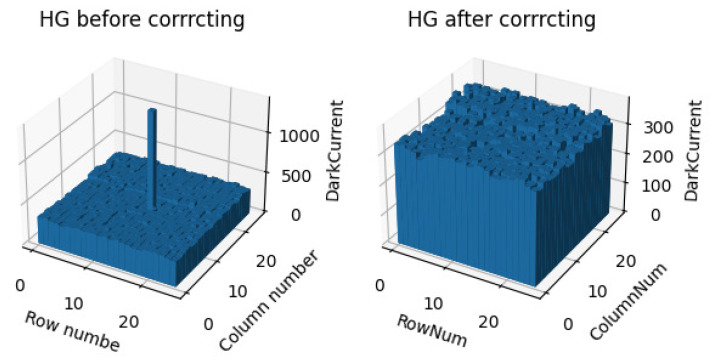
Comparison before and after bad-pixel correction.

When performing a measurement, the detector should first be operated in rated state, and all the lights in the laboratory should be turned off at the same time. The light signal output by the integrating sphere should be blocked by a black-light-shielding plate. After dark-background imaging, there are a few bad pixels on the photodetector that have problems with the manufacturing process. When correcting bad pixels, a 3 × 3 rectangular area with bad pixels is selected as the center to calculate the mean value to replace the bad pixels’ output. A comparison before and after correction is shown in Figure 6. The average value of the dark current measured via the four gains is shown in Table 1. Because of the detector defaulting to performing correlated double sampling (CDS) on the HG, the dark current of the highest-gain HG is relatively low compared with the three other gains. CDS refers to sampling the two levels of the output signal, that is, sampling the reset and signal levels. If the two sampling values are subtracted, the interference of reset noise is basically eliminated. Therefore, HG dark current DN is the smallest.

### 3.2. Determination of the Linear Dynamic Range of Four Gains

Due to the particularity of the pixel-level adaptive-gain imaging method, the overall dynamic range of the system is composed of segmented linear dynamic ranges of the four gains. Therefore, it is necessary to determine the linear dynamic range of the four gains during laboratory calibration in order to judge the value at which the gain is switched. If the ADC quantization bit used for data readout is 14, the upper-limit DN that each gain can output is 214, that is, 16,383. In actual imaging, each gain is saturated when it is close to the maximal output DN, resulting in nonlinear errors. Therefore, it is necessary to measure the maximal linear value of the gains.

In actual measurement, the current controller is used to control the working current of the integrating sphere, so that the integrating sphere can output optical signals of different energy levels. The interval between energy levels should be as small as possible, so that the maximal measured linear value is more accurate. Whether the output value of the photodetector is nonlinear is judged by linear correlation coefficient R between the output radiance of the integrating sphere and the DN. When R is greater than 0.9 and less than 1, it indicates that the point is highly correlated with the straight fitting line. When R is less than 0.9, it indicates that the point had a large nonlinear error, so the last point adjacent to the point is the maximal linear output DN. The calculation formula of the linear correlation coefficient R is shown in Formula (8):

(8) $$R\left(X,Y\right)=\frac{\mathrm{Cov}(X,Y)}{\sqrt{\mathrm{Cov}(X,X)\mathrm{Cov}(Y,Y)}},$$

where Cov(X,Y) is the covariance between the output radiance of the integrating sphere and the photodetector DN, Cov(X,X) and Cov(Y,Y) are their respective variances, and they are shown as Formulas (9) and (10).

(9) $$\mathrm{Cov}\left(X,Y\right)=\frac{\sum_{i=1}^{n}\left(x_{i}-\bar{X}\right)\left(Y_{i}-\bar{Y}\right)}{n-1},$$

(10) $$\mathrm{Cov}\left(X,X\right)=\frac{\sum_{i=1}^{n}\left(X_{i}-\bar{X}\right)\left(X_{i}-\bar{X}\right)}{n-1},\mathrm{Cov}\left(Y,Y\right)=\frac{\sum_{i=1}^{n}\left(Y_{i}-\bar{Y}\right)\left(Y_{i}-\bar{Y}\right)}{n-1},$$

By judging the linear correlation coefficient, the maximal DNs of the four gains’ linear regions are obtained representing the DNs of gain switching. The four gains’ linear regions and switching points are shown in Table 2. During pixel-level adaptive-gain imaging, when the photodetector output DN reaches the linear maximum of the current gain, it switches to the linear region of the next gain.

### 3.3. Measurement of Proportional Coefficient between Adjacent Gains

**Figure 7:**
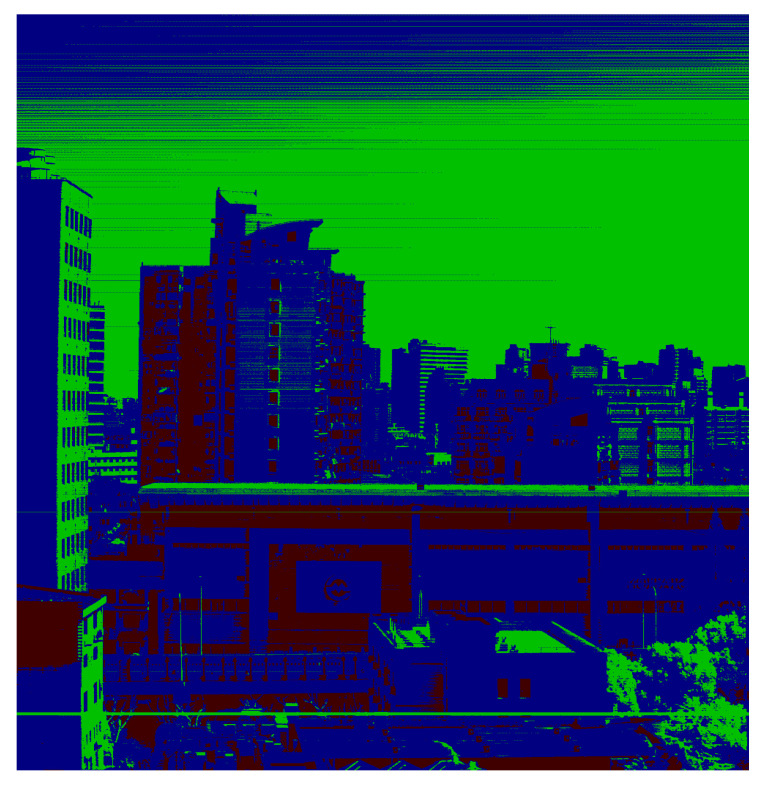
Image of pixel-level adaptive-gain mode.

When pixel-level adaptive-gain mode is used for imaging, pixels on the photodetector use different gains. In Figure 7, the pixel displayed in red is MG, LG in blue, and ULG in green. Before target information extraction, different gains must be converted into the same data standard to connect different gain DNs with the actual target radiance. During gain normalization, it is necessary to measure the conversion relationship among gains and normalize them to the highest gain value. Since the dynamic range span of the four gains is large, the conversion relationship among HG, LG, HG, and ULG cannot be directly measured. Therefore, when calculating the conversion ratio coefficient, only the linear region with the higher gain in the two adjacent gains can be used with a lower gain; lastly, cumulative conversion is performed for the numerical level of the HG. Therefore, the proportional coefficient between two adjacent gains can only be calculated in the linear region with the higher gain in the adjacent gains; then, it is converted into the numerical standard of the highest-gain HG through the relationship between adjacent gains.

**Figure 8:**
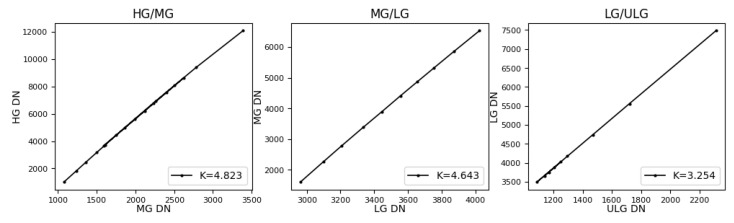
Proportional relationship of adjacent gains.

Under the same lighting conditions, when calculating the proportional relationship between adjacent gains within the linear range of a gain, the DN with a high gain rises faster and reaches saturation first, while the DN with a low gain rises slowly and remains within the linear range. Therefore, one should use the DN with a lower gain as the independent variable, and the DN with a higher gain as the dependent variable, and calculate the proportional relationship between gains through linear fitting as shown in Figure 8.

The calculation process of converting different gains into HG is as follows: assume that the conversion coefficients from MG to HG are k1 and b1, from LG to MG are k2 and b2, and from ULG to LG are k3 and b3. Their values are shown in Table 3. Then, the normalized conversion coefficients from MG to HG are $K_{HM}=k1$ and $B_{HM}=b1$, the coefficients from LG to HG are $K_{HL}=k1\times k2$ and $B_{HL}=k2\times b1+b2$, and the coefficients from ULG to HG are $K_{HU}=k3\times K2\times k1$ and $B_{HU}=k3\times k2\times b1+k3\times b2+b3$.

### 3.4. Determination of Absolute Radiometric Calibration Coefficient

The purpose of absolute radiometric calibration is to establish the quantitative relationship between the payload observation DN and the radiance of the entrance pupil, so that the radiance of the ground target can be retrieved during orbit operation. The quantitative relationship between radiance and the photodetector DN in laboratory absolute radiometric calibration is expressed in Formula (4). The absolute radiometric calibration system is shown in Figure 5.

**Figure 9:**
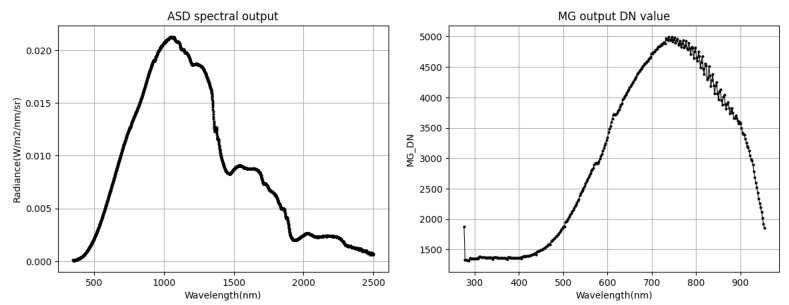
ASD spectral radiance and photodetector spectral response curve under the same light conditions.

In actual detection during orbit, due to grating dispersion splitting, the wavelength of the photodetector’s spectral dimension and the corresponding absolute radiometric calibration coefficient are different. After the spectrometer is installed, the visible light with a spectral range of 375–950 nm is distributed in the spectral dimension of the photodetector with a spectral resolution of 2.16 nm. After nonuniformity correction, the inter-row response of photodetectors remains consistent, and the inter-row response changes with the wavelength of incident light. Figure 9 shows the radiance and detector-response curves of the ASD output under the same lighting conditions. The detector spectral range is redundant. The effective spectral dimension starts at 350 nm and ends at 920 nm, totalling 264 lines. The detector response curve is converted from the spectral dimension serial number into the wavelength. Because the quantum efficiency of the photodetector in the near-infrared channel decreases, the response DN in the near-infrared region decreases.

**Figure 10:**
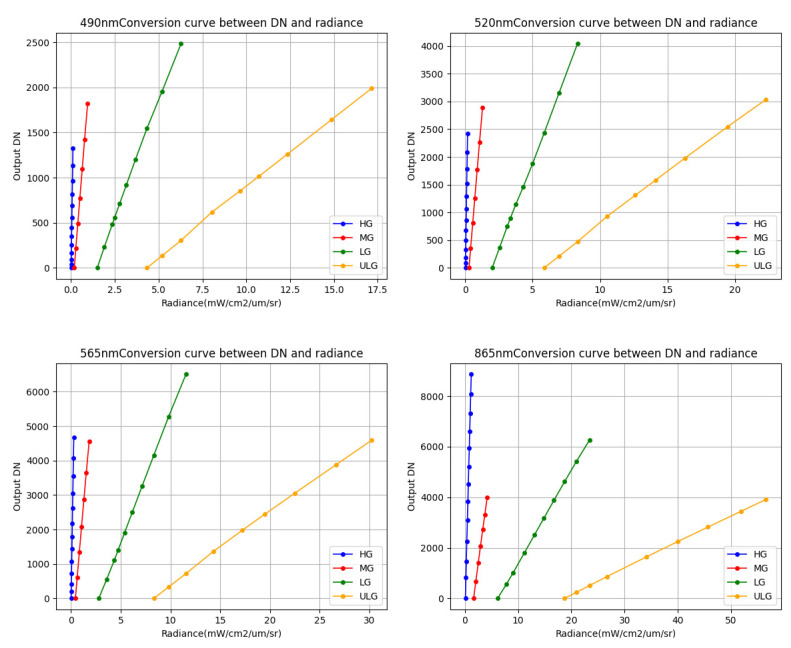
Photodetector response curves of four spectral channels with the radiance detected with ASD as reference.

The absolute radiometric calibration coefficient is calculated for each line of the spectral dimension, and four spectral channels are randomly selected from the results for display. The absolute radiometric calibration curves are shown in Figure 10, and the absolute radiometric calibration coefficient and linear correlation coefficient are shown in Table 4.

### 3.5. Single-Gain Nonlinear Correction

In some specific imaging scenarios, for example, when imaging weak reflectivity targets such as Case 1 waters or targets in dawn or dusk light conditions, high HG or MG is used as a read-out to improve SNR; LG or ULG gear is also selected for the single-gain imaging of sea ice and thick cumulus clouds to avoid signal saturation. When using single-gain imaging, the linearity of each gain becomes the focus of research. In addition, when measuring an on-orbit gain scaling coefficient, it is necessary to read out the four gains’ imaging data at the same time after a single exposure, so that the coefficient can be calculated on the basis of four images of the same target. This requires high performance from each gain.

**Figure 11:**
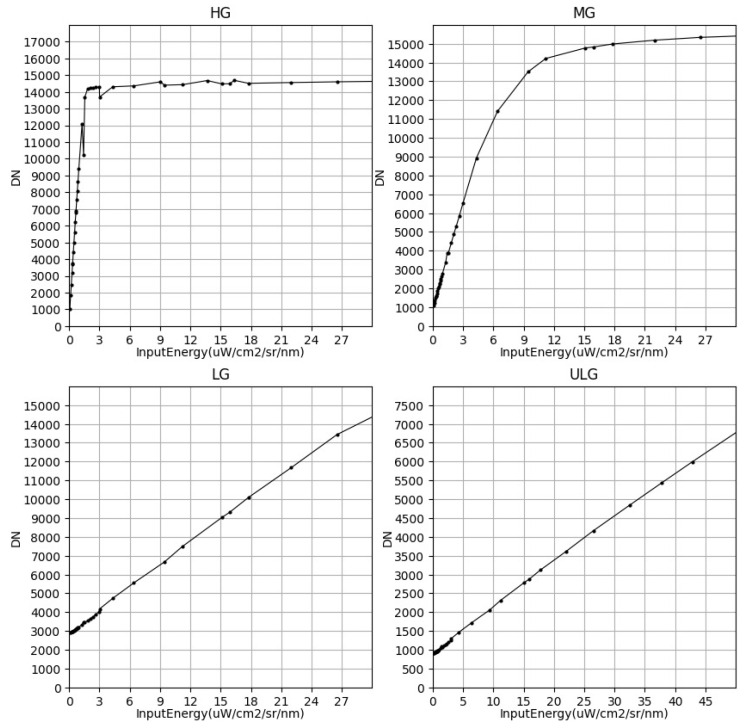
Four gains’ response curve.

In fact, the linear working region of the photodetector is limited, and there are some nonlinear areas at the bottom and top of the response curve. If the nonlinear region is small or the region with large nonlinearity is close to the detector saturation DN, these nonlinear responses do not affect imaging with a single gain. However, if the nonlinear region is large, the output image quality of this gain is greatly reduced. As shown in Figure 11, the response curves of HG, MG, LG, and ULG in single-gain imaging are shown. The nonlinear areas of HG, LG, and ULG are small, which does not cause image quality degradation in single-gain imaging. In contrast, the nonlinear area of MG is large, which causes large nonlinear errors in imaging; this further affects remote-sensing inversion and applications, so the nonlinear correction of the gear is required.

The characteristics of a typical photodetector response curve are that the response DN increases linearly with an increase in input energy at a low energy input; when the input energy exceeds a certain value, the response DN becomes saturated, and a horizontal asymptote appears. For such characteristics, a polynomial model cannot be used to fit the overall response curve because, although a polynomial of a sufficiently high order can fit the response curve from zero input to saturation, if the linear rise at low energy and the asymptotic saturation at high energy are to be met simultaneously, the fitting polynomial must produce curve oscillation in the saturated region. Therefore, a piecewise method is proposed to fit the absolute MG radiometric calibration response curve. Linear fitting is used for the linear growth region with a low energy input; a third-order polynomial is used to fit the region where te linearity decreases with a slow growth rate but is not yet saturated; the presaturation and saturation regions are replaced by a fixed value due to the loss of the input–output relationship. The equation is as follows:

(11) $$y=\left\{\begin{array}{c} k_{f}x+b_{f}\\ a_{0}x^{3}+a_{1}x^{2}+a_{2}x+a_{3}\\ y_{sat} \end{array}\right.,$$

where *y* is the detector response DN; *x* is the radiance emitted by the integrating sphere during laboratory radiometric calibration; $k_{f}$ and $b_{f}$ are the slope and offset during linear fitting, respectively, and *a*_0_, *a*_1_, *a*_2_, *a*_3_ are the coefficients of third-order curve fitting. The goodness of fit of the linear and third-order polynomial fittings in piecewise fitting is 0.9984 and 0.9996, respectively, and the fitting effect is excellent. The calculation formula of the goodness of fit is shown in Equation (12).

(12) $$GOF=\frac{\sum_{i=1}^{n}{\left({\hat{y}}_{i}-\bar{y}\right)}^{2}}{\sum_{i=1}^{n}{\left(y_{i}-\bar{y}\right)}^{2}},$$

where $y_{i}$ is the detector response DNs at different energy levels of the integrating sphere, ${\hat{y}}_{i}$ is the fitting value of the detector response at different energy levels, and $\bar{y}$ is the average of all response DNs.

**Figure 12:**
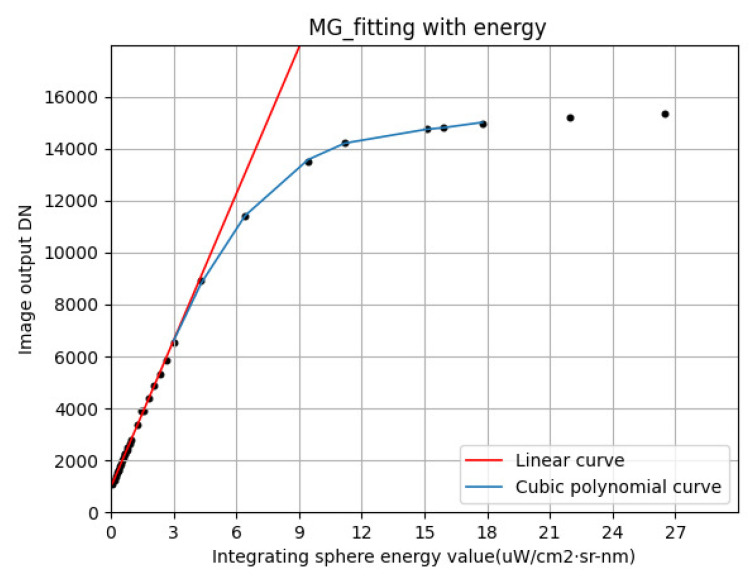
Fitting curve of the laboratory absolute radiometric response of MG.

MG’s laboratory absolute radiometric response points and the fitting curves fitted by linear and third-order curves are shown in Figure 12. Nonlinear correction is needed for a nonlinear region fitted by a third-order polynomial. Since the sampling point for establishing a response curve model is the average value of the photodetector image at different input energy levels, it is necessary to conduct nonuniform correction on each point before nonlinear correction on the MG image. The MG linear response region is selected as the reference value for nonuniform correction. After nonuniform correction, the piecewise fitting model can be used to correct MG images.

**Figure 13:**
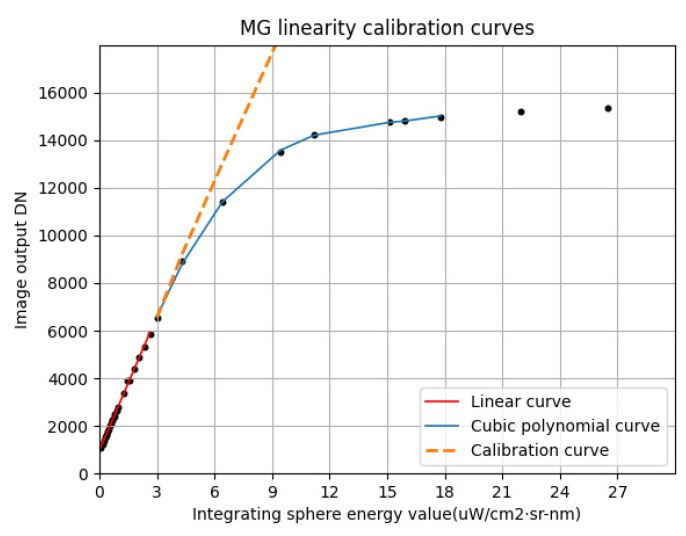
Response curve after nonlinear correction.

The goodness of fit of the third-order polynomial in the nonlinear MG response region is 0.9996, which means that it could be approximately considered that the third-order polynomial could completely replace the response curve in the nonlinear region. The third-order polynomial fitted in this region could be bidirectionally mapped with the linear equation in the linear area, thus establishing a mapping function. The mapping function of the third-order polynomial and the linear equation can be obtained by eliminating the input energy x of the integrating sphere through formula derivation. The mapping equation is as follows:

(13) $$g=a_{0}{\left(\frac{y_{f}-b_{f}}{k_{f}}\right)}^{3}+a_{1}{\left(\frac{y_{f}-b_{f}}{k_{f}}\right)}^{2}+a_{2}\left(\frac{y_{f}-b_{f}}{k_{f}}\right)+a_{3},$$

where $y_{f}$ is the DN obtained by calculating and fitting the input energy of the nonlinear region through the linear equation; *g* represents the DN fitted by the third-order polynomial in the nonlinear region, which can be approximately regarded as the real value through the above discussion. Through the inverse function of Formula (8), the DN of the third-order polynomial can be converted into a linear DN under the same input energy to perform nonlinear correction. Figure 13 shows the response curve of the nonlinear area after correction.

## 4. Conclusions

In order to meet the design requirements of high sensitivity and a high dynamic range, many remote-sensing loads are set with multistage gain, but usually with only one or two gains, and gain switching is performed for the whole frame or column. A pixel-level adaptive-gain imaging system exposes a pixel once and reads out four times with different gains, so that the dynamic range of the instrument is significantly expanded under the condition of ensuring a high SNR. However, the laboratory radiometric calibration process corresponding to this new imaging system is quite different from conventional instruments. This paper analyzed and realized the absolute radiometric calibration requirements for the two imaging modes of a pixel-level adaptive-gain imaging system: single-block-gain imaging and adaptive-gain imaging:

- The dark current of the photodetector is measured and the bad pixels that have process problems are corrected.
- In pixel-level adaptive-gain imaging mode, the linear dynamic range of the four gains is measured and used as the switching standard to form the overall linear dynamic range of the system.
- The proportional relationship between adjacent gains is obtained within the linear range of each gain to facilitate normalized image processing after adaptive-gain imaging.
- A laboratory stable integrating sphere is used to measure the absolute radiometric calibration coefficient and calibration offset of the four gains, and the ASD is used to measure the radiance of the integrating sphere as a reference for calibration to establish the quantitative relationship between the radiance and the observation value of the imaging system.
- In some scenes, the single-gain imaging mode of an imaging system is used, so this paper corrected the nonlinear region of MG, the gain with the largest nonlinear error, to prevent the nonlinear error from affecting the imaging.

In this paper, the parameters of four gains’ radiance inversion linear equations were calibrated, the switching point between the adjacent gains was found and the linear dynamic range corresponding to each gain was determined, and the conversion relationship between the four gains was established, which lay the foundation for subsequent pixel-level adaptive-gain image fusion. Laboratory radiometric calibration establishes the relationship between instrument detection values and actual physical parameters, which is the first step in the quantitative application of remote-sensing images. After the laboratory radiometric calibration process in this paper is completed, the basic parameters of the pixel-level adaptive-gain imaging system could be determined, which can provide reference for this imaging system for on-orbit operation, and assist in the application of remote-sensing images obtained from on-orbit detection.

## Figures and Tables

**Table 1:** Average value of four gains’ dark current.

Gain Level	Average Dark Current DN
HG	335.58
MG	1281.51
LG	1179.44
ULG	1183.30

**Table 2:** Four gains’ linear regions and switching points.

Gain Level	Switching Point DN	Linear Region
HG	14,186	336∼14,186
MG	11,413	1282∼11,413
LG	13,254	1180∼13,254
ULG	Nought	1184∼13,411

**Table 3:** Four gains’ conversion coefficients between adjacent gains.

Adjacent Gains	Conversion Slope	Conversion Offset
HG/MG	4.82	−128.68
MG/LG	4.64	436.17
LG/ULG	3.25	−152.71

**Table 4:** Absolute radiometric calibration coefficients of four spectral channels with HG, MG, LG, and ULG.

Central Wavelength	Gain Level	Calibration Slope	Calibration Offset	Correlation Coefficient
490 nm	HG	0.000080	−0.025551	0.9998
	MG	0.000416	−0.563583	0.9999
	LG	0.001922	−2.573559	0.9995
	ULG	0.006441	−8.027152	0.9997
520 nm	HG	0.000066	−0.021217	0.9998
	MG	0.000347	−0.477446	0.9999
	LG	0.001582	−2.158486	0.9998
	ULG	0.005402	−6.947947	0.9997
565 nm	HG	0.000057	−0.019258	0.9999
	MG	0.000302	−0.435559	0.9998
	LG	0.001332	−1.797282	0.9999
	ULG	0.004765	−6.713449	0.9996
865 nm	HG	0.000124	−0.055145	0.9998
	MG	0.000638	−0.959038	0.9999
	LG	0.002724	−3.587031	0.9999
	ULG	0.009654	−13.074396	0.9999

## Data Availability

The study did not report any data.
